# Transparent Fingerprint Sensor System for Large Flat Panel Display

**DOI:** 10.3390/s18010293

**Published:** 2018-01-19

**Authors:** Wonkuk Seo, Jae-Eun Pi, Sung Haeung Cho, Seung-Youl Kang, Seong-Deok Ahn, Chi-Sun Hwang, Ho-Sik Jeon, Jong-Uk Kim, Myunghee Lee

**Affiliations:** 1 Ulsan National Institute of Science and Technology, School of Electrical & Electronic Engineering, Ulsan 44919, Korea; stepkr@unist.ac.kr; 2 Electronics and Telecommunications Research Institute, Reality Device Research Division, Daejeon 34129, Korea; jepi@etri.re.kr (J.-E.P.); helloshcho@etri.re.kr (S.H.C.); kang2476@etri.re.kr (S.Y.K.); lovesky@etri.re.kr (S.-D.A.); hwang-cs@etri.re.kr (C.-S.H.); 3 CrucialTec, Pankyo 13486, Korea; hsjeon@crucialtec.com (H.-S.J.); jukim@crucialtec.com (J.-U.K.)

**Keywords:** fingerprint, transparent, a-IGZO thin film transistors (TFTs), sensor array, readout integrated circuit (ROIC)

## Abstract

In this paper, we introduce a transparent fingerprint sensing system using a thin film transistor (TFT) sensor panel, based on a self-capacitive sensing scheme. An armorphousindium gallium zinc oxide (a-IGZO) TFT sensor array and associated custom Read-Out IC (ROIC) are implemented for the system. The sensor panel has a 200 × 200 pixel array and each pixel size is as small as 50 μm × 50 μm. The ROIC uses only eight analog front-end (AFE) amplifier stages along with a successive approximation analog-to-digital converter (SAR ADC). To get the fingerprint image data from the sensor array, the ROIC senses a capacitance, which is formed by a cover glass material between a human finger and an electrode of each pixel of the sensor array. Three methods are reviewed for estimating the self-capacitance. The measurement result demonstrates that the transparent fingerprint sensor system has an ability to differentiate a human finger’s ridges and valleys through the fingerprint sensor array.

## 1. Introduction

Fingerprint sensors have become a popular biometric identification solution for mobile devices, such as the smart phone [1,2]. There are many technologies for realizing a fingerprint sensor in terms of sensing scheme: Capacitive [3,4], optical [5], or ultrasonic sensors [6]. Thus far, fingerprint sensor modules for mobile phones are mainly a small form factor design. Lately, there is an effort to adopt the entire display area of a smart phone as a fingerprint sensor. In such cases, a transparent fingerprint sensor should be embedded under the cover glass on top of an existing display panel area, in order to realize a front-panel fingerprint system.

A sensor panel’s pixel dimension must be less than 50 μm × 50 μm to realize a high-resolution fingerprint sensor array. Such high-resolution dimensions are required to ensure security when verifying a person’s identity. A pixel with self-capacitive type sensing, which consists of a readout switching TFT (thin film transistor) and a conductive plate, has a strong advantage, as it operates with a normal display control scheme and easily realizes high PPI (pixel per inch).

**Figure 1:**
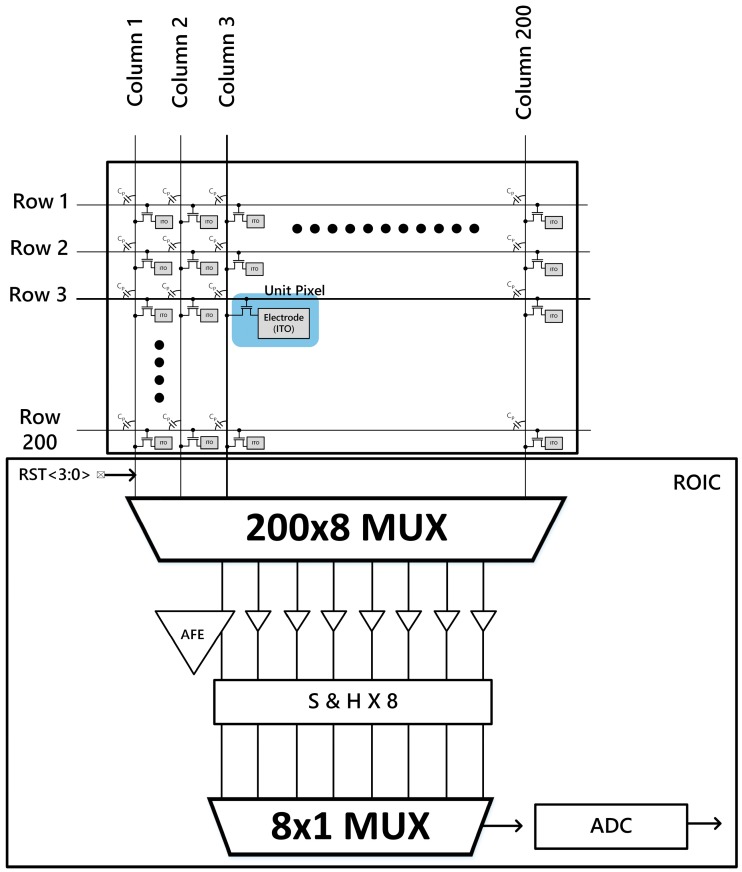
Block diagram of the proposed finger print sensor system.

In order to realize large panel sensor systems, there are a few technical challenges to overcome. In this case, a sensor relies on self-capacitance, which is formed between a human finger and sensing electrodes. First, the sensing area is inversely proportional to the resolution of the sensor pixel array. Second, the capacitance difference caused by the ridges and valleys of a fingerprint would be unrealistically small, since mobile phones often require a thicker cover glass for safety. Furthermore, a given cover glass material also affects the permittivity of capacitance. In this paper, a transparent fingerprint sensing system, as shown in Figure 1, is introduced. The sensor panel array achieves more than 500 PPI for a finer resolution. In addition, a low-power read-out integrated circuit (ROIC) is implemented for supporting a large panel sensor array.

## 2. Structure of a Transparent Sensor Panel

### 2.1. Structure of a Proposed Fingerprint Sensor Pixel

**Figure 2:**
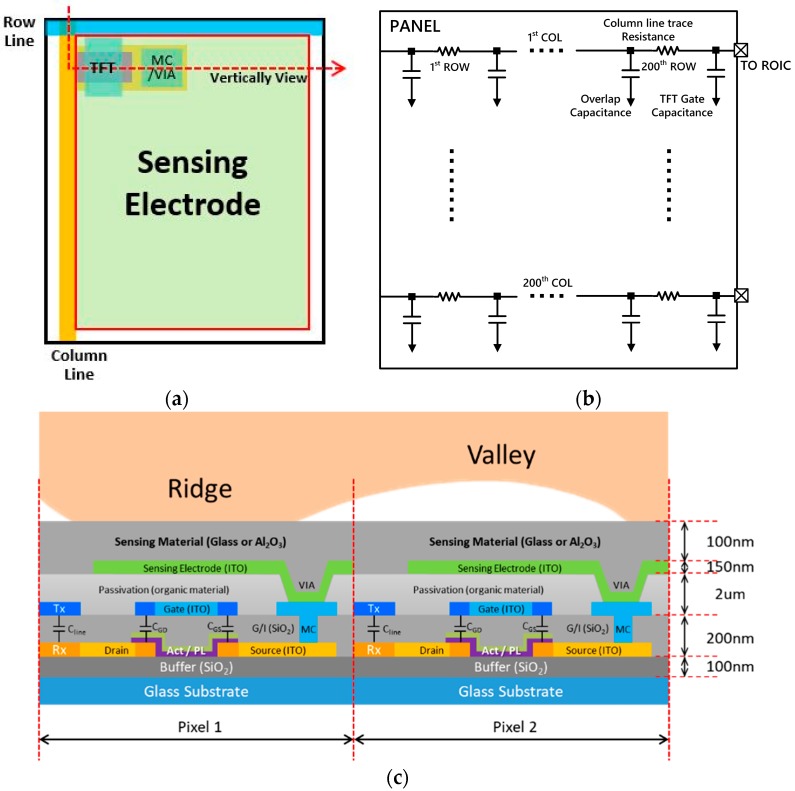
(**a**) Top view of the self-capacitance type sensor pixel structure; (**b**) equivalent parasitic model of the sensor array; and (**c**) cross-sectional view of the proposed sensor pixel.

Figure 2 shows the pixel structure of the proposed sensor panel array. Figure 2a shows the structure of the unit pixel. The pixel is composed of a read-out switching TFT and a sensing electrode for measuring the self-capacitance between the finger and the sensing electrode. Figure 2b illustrates the electrical model for the parasitic resistances and capacitances of the sensor panel. Figure 2c depicts a cross-section of the sensor pixels. An amorphous-Indium gallium zinc oxide (a-IGZO) semiconductor is used for the active layer, and indium tin oxide (ITO) for both the bus line and the sensor electrode, are used to achieve a higher transparency.

Reportedly [7], the optical bandgap (3.05 eV) of the a-IGZO semiconductor ensures high transparency compared to conventional a-Si semiconductors (1.6 eV). [8] The transmittance of the fabricated TFT array is about 75% (measured) with a reference glass of 90% transmittance. Since the transparent fingerprint sensor array can be attached to a conventional display panel to detect identity, the display bezel area is drastically minimized by removing the area of the opaque fingerprint sensor array and its control circuit. The fingerprint sensorcan also be used as a multi-fingerprint touch screen for the entire display panel area. As shown in Figure 2c, the thick organic buffer layer (~2 μm) on the sensing electrode is used to avoid interference between a finger and the bus line before the TFT process. The driving method of the fingerprint sensor array is like that of a conventional active-matrix TFT display panel. The gate or row line is driven by a commercial gate driver integrated circuit (IC).

### 2.2. Calculating the Self-Capacitance of the Pixel Structure

**Figure 3:**
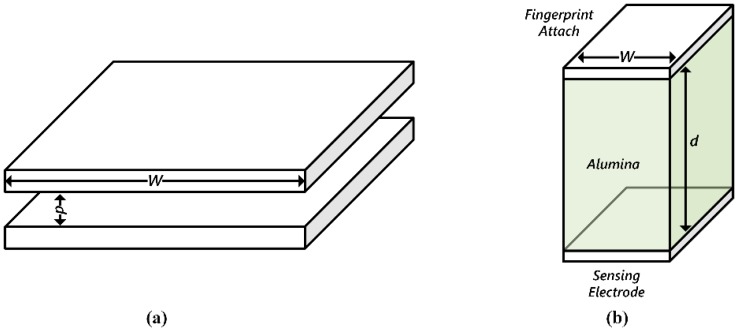
(**a**) Simple capacitor structure for an infinitesimal gap; and (**b**) the capacitor structure for a fingerprint sensor case.

Since the proposed sensor panel relies on self-capacitance sensing, it is critical to have an accurate estimation of the capacitance between a finger and an electrode of each pixel. For the proposed fingerprint sensor pixel, as shown in Figure 2c, the gap, *d*, between the finger and the sensor electrode of each pixel is bigger than the side dimension, *W*, of the electrode plate. In the case of *d* << *W,* in Figure 3a, the capacitance value is easily estimated using a simple capacitance equation (Equation (1)). However, the proposed sensor pixel electrode is 44 μm × 44 μm, and the thickness of the cover glass, or passivation layer, is 100 μm. In other words, the gap between the electrode and the finger is at least twice the width of one of the electrode’s sides [9]. Under the condition where *d* >> *W*, as shown in Figure 3b, Equation (1) is no longer valid, and another form of equation is needed to estimate the capacitance value.

C = ε (A/*d*) (1)

Another issue is the close proximity among neighboring pixels, which cause an interference in the electric field. Two approaches are considered to estimate the capacitance between the finger and the pixel electrode of the sensor panel: BEM (Boundary Element Method) [10] and a commercial simulator tool [11]. The capacitance estimation using BEM does not include the effects of interference from neighboring pixels. Thus, the commercial simulator is used to estimate the capacitance between the finger and pixel electrode. Parasitic capacitance caused by the sensor panel is also carefully estimated based on the dielectric constant of the material and the distance between each pixel.

### 2.3. Fingerprint Capacitance Modeling

**Figure 4:**
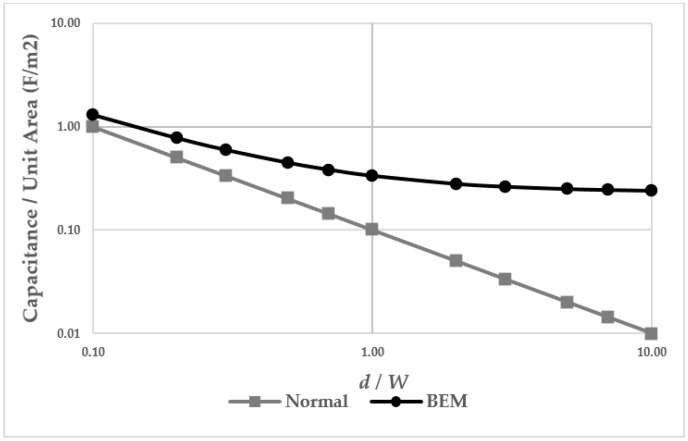
Capacitance value: Ideal parallel-plate calculation vs. BEM.

Accurate estimation of the capacitance values from the sensor is critical for achieving good sensitivity in the ROIC design. Equation (1) is for an ideal infinitesimal parallel plates and valid only for *d* << *W*. BEM helps to estimate the capacitance under the condition of *d* >> *W*. This method takes into account the electrostatic field and charge density under the condition. As demonstrated in Reference [9], Figure 4 shows the normalized capacitance value between an ideal infinitesimal plate capacitance, based on Equation (1), and the capacitance using BEM. The *Y*-axis shows the normalized value to the ratios of *d* and *W* for easy comparison between the two methods. As *d* is smaller, the normalized constant approaches Equation (1). As *d* increases, the estimated capacitance value using the two methods differs. Table 1 shows a deducted capacitance value comparison between Equation (1) and BEM. The values represent the capacitance value by unit pixel.

However, the effect of neighboring pixels is not reflected in the BEM capacitance result. A commercial simulator was used in order to take circumferential electrical interference effects.

**Figure 5:**
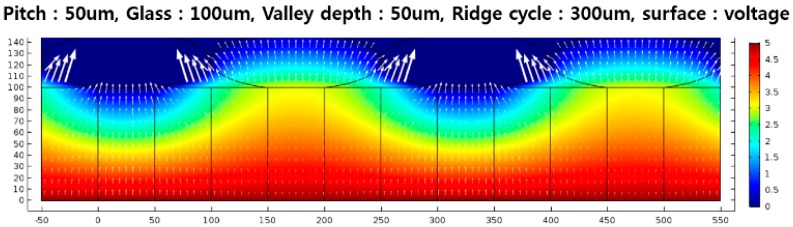
The contour plot of the electric field in the sensor structure in the horizontal direction.

The electrical field simulation of the sensor array helps to estimate the finger capacitance with accuracy. To obtain the detailed electrical information in Figure 5, the structural parameters, such as the sensor pitch, ridge cycle and valley depth, are considered. A glass (ε_r_ = 7.3) of 100 μm in thickness was chosen as a cover glass. In Figure 5, a vertical view of the electric fields of the sensor pixel is shown as a contour plot. The bottom-sensing electrode (deep red area) voltage was set to 5 V and the surface voltage of the finger (deep blue area) to 0 V, assuming the human body’s voltage is grounded. The simulation results show that charges on ridges to the sensor electrode are larger than those of the valleys to the sensor electrode.

**Figure 6:**
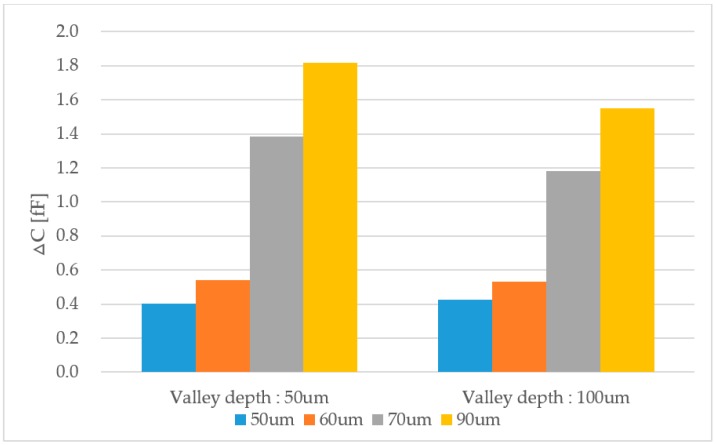
The simulation results on the capacitance difference (∆C) of the ridge to valley region (valley depth: 50 μm and 100 μm, ridge pitch: 300 μm, and the pixel pitch: 50/60/70/90 μm).

Figure 6 shows the simulation results with the various pixel pitches. The sensing capacitance is proportional to the pixel pitch size. The data show a significant capacitance increase when the sensor pitch is more than 70 μm. For the effect of valley depth (50 μm and 100 μm), the valley depth of 50 μm shows a larger capacitance difference than that with a 100-μm valley depth, because of increased ridge areas with a low valley depth. However, a similar capacitance difference at a smaller pixel pitch (50 μm and 60 μm) is calculated. To obtain a uniform capacitance difference with various valley depths, we assume that the pixel pitch must be designed to be as small as the contact area of a human fingerprint ridge. For this reason, although the 90-μm sensor pitch showed the largest capacitance difference, a 50-μm pixel pitch was chosen for the sensor array design, which also has a better resolution.

The resistance of the bus line (row and column lines) of the sensor panel array, the line capacitance (C_line_), and sensor array dimensions were designed, as shown in Table 2. The measured electrical characteristics of the a-IGZO TFTs, such as threshold voltage, subthreshold slope (SS) and field-effect mobility, are 1 V, 250 mV/dec and 10 cm^2^/V∙s, respectively. The comparison of the self-capacitance based on 3 methods is shown in Table 3.

By taking the surrounding electrical interference effects into account, each pixel shows a 24% increase in capacitance value compared to the estimation using BEM as shown in Table 3.

## 3. ROIC Structure

### 3.1. AFE Structure

ROIC takes 200 channel inputs from the sensor panel and feeds those inputs into a 200 × 8 multiplexer, followed by eight analog front-end (AFE) low-noise amplifiers, and a sample and hold circuit (S&H). A successive approximation analog-to-digital converter (SAR ADC) converts those analog signals into digitized values, which correspond to the voltage values of a fingerprint’s ridges or valleys. By using a smaller number of AFEs, the power dissipation and the size of the ROIC are minimized.

The AFE input stage of ROIC should be able to sense the capacitance difference between the fingerprint (ridge or valley area) and the sensor electrode when a user’s finger touches the cover glass on a sensor pixel area. Designing a highly-sensitive ROIC for sensing the proposed sensor array is required. While there are relatively large parasitic capacitances, contributed by the routing metal wires of the sensor panel, sensing capacitances for each pixel is very low due to the gap between the finger and pixel’s electrode. The AFE input stage consists of a charge-sharing stage and a current output generation stage using a differential input mode. [12,13,14,15]

**Figure 7:**
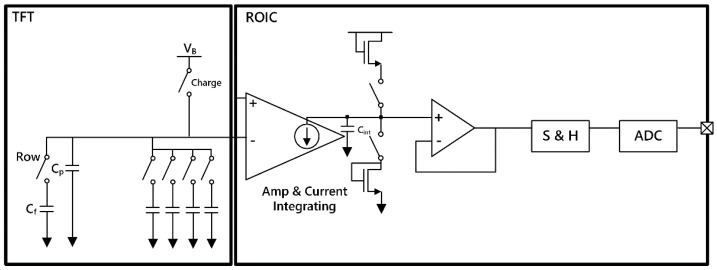
A pixel equivalent circuit model and a single channel analog front end (AFE) of fingerprint senor system.

**Figure 8:**
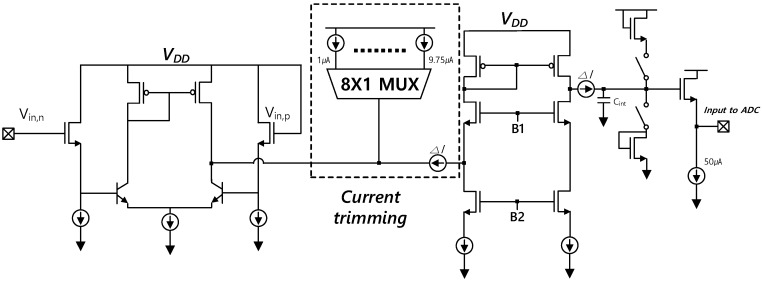
Structure of readout integrated circuit (ROIC)’s charge to current converter.

As shown in Figure 7, the electrical behavior of the fingerprint sensing system can be modeled in two parts: the TFT sensing array and the analog-front-end (AFE) of ROIC. The TFT sensing array shows parasitic capacitances and a charge-sharing scheme. The AFE stage consists of a charge-to-current converter, a buffer, and a sample and hold circuit. Figure 8 illustrates a differential amplifier stage that is used as a charge-to-current converter. The amplifier’s differential inputs have a bias or reference voltage at the positive input, and the voltage associated with charge sharing from the TFT at the negative input. The current output from the converter is proportional in magnitude to the input voltage difference of the amplifier inputs. The voltage of the negative input side of the amplifier is expressed as Equation (2), and the current from the converter is expressed as Equation (3).

V*_in,n_* = (C_p_/(C_p_ + C_f_))·V_ref_ (2)

∆I = g_m_·(V_ref_ − V*_in,n_*), (3)

where C_p_ represents a parasitic capacitance of the panel, C_f_ is the finger capacitance of each pixel, and g_m_ is the transconductance of the bipolar transistor.

The output current, amplified by the current mirror stage, charges the integrating capacitance, C_int_, to a voltage output. To avoid saturation of the voltage output, the current trimming block, which helps control output current, can be used. Capacitance error or external interference can cause over-current. However, the current trimming block can prevent the saturation caused by excessive incoming current.

### 3.2. ROIC Signal Processing Sequence

Figure 1 shows the overall block diagram of the transparent fingerprint system. When the sensor array panel is assembled with the ROIC, 200 channels from the sensor panel are connected to eight AFE amplifiers through a 200 × 8 (or eight sections of 25 × 1 MUX) multiplexer, which is sequentially selected using RST<3:0> signals within the ROIC. Only eight analog-front-end (AFE) stages simultaneously read the capacitance value from the fingerprint sensor (200 columns). The designated scan time for each row is broken into 25 subdivisions in order to read all 200 channels in sequence. This helps to significantly reduce the power dissipation of ROIC.

**Figure 9:**
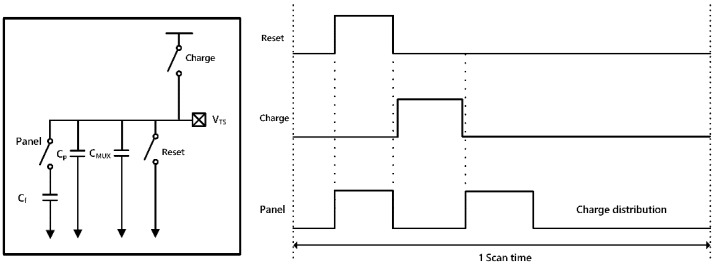
Timing diagram for data processing sequence.

During each row’s 0.1 ms scan time, there are three steps of data processing: Charge, reset, and charge sharing. Figure 9 shows the timing diagram. During the reset period, the parasitic and fingerprint capacitances are set to an initial state. This is in preparation for charge sharing. The fingerprint capacitance is much smaller than the parasitic capacitance, and the remaining charge dramatically affects the output voltage after charge sharing.

Next is the pre-charging to the parasitic capacitance, when the panel switch remains off, and the charge switch turns on. The fingerprint capacitance remains the reset condition, but the parasitic capacitors (C_P_ and C_MUX_ in Figure 9) are charged with the preset bias voltage. During the following charge sharing period, the panel switch turns on and the charges in the parasitic capacitors are shared with the capacitor from the fingerprint sensor, C_F_. The voltage level of V_TS_ in Figure 9 drops from the preset bias. Both capacitances have the same potential. From Equation (2), the voltage (V*_in,n_*) is compared to the reference voltage (or V*_in,p_*) and the difference in voltages indicates whether it is a ridge or a valley. Table 4 shows the voltages during each sequence.

To get enough gain, a bipolar transistor pair (β = ~50) is used, as shown in Figure 8. Table 4 shows the voltage values at the differential input nodes during each sequence. The output voltage is integrated at the integrating capacitor (C_int_) by the charge-to-current converter. Equation (4) shows the output voltage.

∆V_out_ = G_m_·∆ (V*_in,p_* − V*_in,n_*)·*t*/C_int_ (4)

Bipolar transistors and a high current source are used to get higher transconductances. The following parameter values in Equation (4) are used: G_m_ = ~0.001, ∆(V*_in,p_* − V*_in,n_*) = 150 μV, *t* = 10 μs, and C_int_ = 2 pF. Thus, the difference in output voltage between ridges and valleys is around 0.75 V.

**Figure 10:**
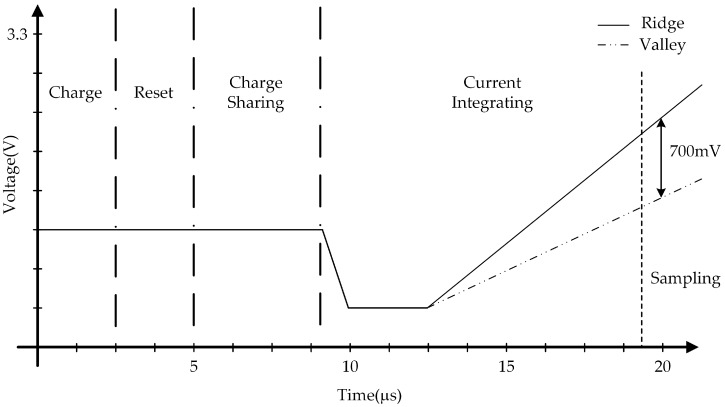
Simulation result of the AFE stage.

**Figure 11:**
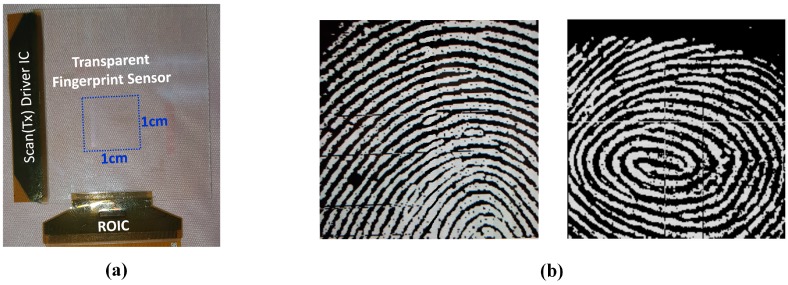
(**a**) The assembled module of the transparent fingerprint sensing system. ROIC is packaged on COF (chip-on-film) and attached to the panel; (**b**) measured fingerprint image with pores.

Figure 10 shows the simulation results of the one channel block. The voltage, which is integrated over capacitance, goes into the buffer. The buffer delivers the voltage to the sample and hold circuit. The sample and hold circuit selects the voltage value of the slope. The delivered sampled voltage is converted into digital signals by an SAR ADC. At the end of the scan time, the registers of the SAR ADC save the digitized output voltage level of the sensor’s individual pixel. The ADC output data would are displayed with greyscale between black and white colors in order to show the fingerprint images on a separate display screen, as in Figure 11b.

## 4. Measurement Results

**Figure 12:**
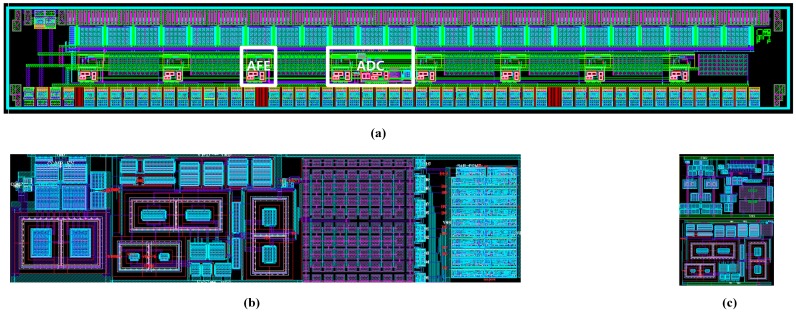
(**a**) ROIC layout; (**b**) ADC sub-block, (**c**) AFE sub-block.

Figure 11 shows the sensor panel assembly with ROIC, and the measurement results of the transparent fingerprint sensor system. With the proposed transparent fingerprint system, a clear fingerprint image was obtained, as shown in Figure 11b. The white and black colors shown in the figure represent the ridges and valleys of a fingerprint, respectively. Figure 12 shows the layout of the ROIC chip and the function blocks. The area of the entire chip is 5560 × 720 μm^2^, even though the actual active circuit area is much smaller. This is due to the requirements for the number of input pads.

## 5. Conclusions

A transparent fingerprint sensor system, which has a 200 × 200 matrix array with 500+ PPI (pixel per inch), was successfully demonstrated. The key specifications of the system are summarized in Table 5. A custom ROIC, which has a very low power consumption and high sensitivity, was also designed to support the sensor system. The measurement results showed clear fingerprint images, including pores.

## Figures and Tables

**Table 1:** Estimated self-capacitance of the sensor array based on the ideal parallel-plate equation and BEM.

By Equation (1)	By BEM (*d*/*W* = 1.5)
171 *aF*	513 *aF*

**Table 2:** The designed sensor array information.

Pixel Information			Sensor Array Information			
Unit Pixel Dimension	Sensing Electrode Dimension	Resolution	Sensor Array Dimension	PPI	Column Line Resistance	Column Line Capacitance
50 μm × 50 μm	44 μm × 44 μm	200 × 200	1 cm × 1 cm	508	66 kΩ	0.3 pF

**Table 3:** Comparison of the self-capacitance based on 3 methods: Equation (1), BEM, and the commercial tool.

Equation (1)	BEM	Commercial Tool
171 *aF*	513 *aF*	400 *aF*

**Table 4:** The value of V*_in,n_* and V*_in,p_* during the data-processing sequence.

Sequence	V*_in,n_*	V*_in,p_*	V*_in,p_* − V*_in,n_*	∆(V*_in,p_* − V*_in,n_*) (Ridge-Valley)
Charge	0	V_ref_	3.3 V	-
Reset	V_ref_	V_ref_	0 V	-
Charge Sharing	$\frac{C_{p}}{C_{p}+C_{f}}V_{ref}$	V_ref_	300 μV	150 μV

**Table 5:** Specification of the fingerprint sensing system.

	Parameter	Specification
Sensor System	Sensor Array	200 × 200
	Pixel Area	50 × 50 μm^2^
	Electrode Plate Size	44 × 44 μm^2^
	Total Scan time/frame	500 ms
ROIC	Power Supply	3.3 V
	Power Dissipation	9 mW
	Process Technology	180 nm Magnachip
	Chip Area	5560 × 720 μm^2^
